# Challenges and opportunities in the continuity of care for hypertension: a mixed-methods study embedded in a primary health care intervention in Tajikistan

**DOI:** 10.1186/s12913-019-4779-5

**Published:** 2019-12-03

**Authors:** Adanna Chukwuma, Estelle Gong, Mutriba Latypova, Nicole Fraser-Hurt

**Affiliations:** 1 Health, Nutrition, and Population Global Practice, World Bank Group, Washington, DC, 20433 USA; 2 Mount Sinai Health System, New York, NY 10019 USA

**Keywords:** Cascade of care, Continuity of care, Hypertension, Tajikistan, Implementation research

## Abstract

**Background:**

Hypertension, a significant risk factor for ischemic heart disease and other chronic conditions, is the third-highest cause of death and disability in Tajikistan. Thus, ensuring the early detection and appropriate management of hypertension is a core element of strategies to improve population health in Tajikistan. For a strategy to be successful, it should be informed by the causes of gaps in service delivery and feasible solutions to these challenges. The objective of this study was to undertake a systematic assessment of hypertension case detection and retention in care within Tajikistan’s primary health care system, and to identify challenges and appropriate solutions.

**Methods:**

Our mixed methods study drew on the cascade of care framework to examine patient progression through the recommended stages of hypertension care. We triangulated data from household surveys and facility registries within Tajikistan’s Health Services Improvement Project (HSIP) to describe the cascade. Focus group discussions with local HSIP stakeholders identified the barriers to and facilitators for care. Drawing on global empirical evidence on effective interventions and stakeholder judgments on the feasibility of implementation, we developed recommendations to improve hypertension service delivery that were informed by our quantitative and qualitative findings.

**Results:**

We review the results for the case detection stage of the cascade of care, which had the most significant gaps. Of the half a million people with hypertension in Khatlon and Sogd Oblasts (administrative regions), about 10% have been diagnosed in Khatlon and only 5% in Sogd. Barriers to case detection include misinformation about hypertension, ambiguous protocols, and limited delivery capacity. Solutions identified to these challenges were mobilizing faith-based organizations, scaling up screening through health caravans, task-shifting to increase provider supply, and introducing job aids for providers.

**Conclusions:**

Translating findings on discontinuities in care for hypertension and other chronic diseases to actionable policy insights can be facilitated by collaboration with local stakeholders, triangulation of data sources, and identifying the intersection between the feasible and the effective in defining solutions to service delivery challenges.

## Background

Tajikistan has achieved significant success in reducing mortality from acute illness resulting in an increase in life expectancy at birth from 63.1 years in 1990 to 71.2 years in 2017 [1]. However, the burden of non-communicable diseases (NCDs) is increasing. Compared to 1990, when lower respiratory diseases and diarrhea caused the most deaths, ischemic heart disease was the leading cause of death in Tajikistan in 2017, increasing in its contribution to the share of premature mortality by 44% from 2007 [2]. High blood pressure (BP), a risk factor for ischemic heart disease and other chronic conditions, was the third-largest driver of all-cause death and disability in 2017 [2]. Therefore, ensuring the early detection and appropriate management of hypertension can contribute towards reducing death and disability from NCDs in Tajikistan.

However, there are significant gaps in hypertension detection and management in Tajikistan. In a 2013 survey of male and female adults, above 18 years, in Tajikistan by the World Bank, only 10% of hypertension cases had been diagnosed, and 42% of respondents had ever had their BP measured. Furthermore, only 10% of diagnosed cases had attained BP control, indicating that their management could be improved [3]. In a nationally-representative survey of women aged 15 to 49 years carried out 4 years later in 2017, these challenges persisted. Only 17% of respondents with hypertension were aware of their condition and actively managed their BP, while 60% were unaware of their hypertensive status [4]. Cases of undiagnosed and inappropriately-managed hypertension represent missed opportunities to address the growing burden of cardiovascular diseases in Tajikistan. Hence, the Government of Tajikistan and other stakeholders have invested in policies and programs to address gaps in service delivery for hypertension.

In Tajikistan’s National Health Strategy for 2010–2020, there is a clear focus on decreasing the burden of NCDs by increasing preventive activities and promoting proactive management at the primary health care (PHC) level [5]. Furthermore, the NCD Strategy for 2013–2023 aims to reduce the prevalence of hypertension by 3 to 5% between 2017 and 2023 [6]. There are also national strategies focused on population health measures to reduce the risk for cardiovascular diseases and hypertension, by promoting physical activity and healthy eating, and to facilitate the appropriate management of complications of hypertension. Furthermore, Tajikistan has national clinical guidelines for hypertension diagnosis and management at the PHC level, and prescriptions have been oriented towards generic drugs by a ministerial order [7].

There have also been concerted efforts to translate these policies into practice. Significantly, since 2013, in partnership with the World Bank, the Tajikistan Ministry of Health and Social Protection (MoHSP) has implemented results-based financing (RBF) and collaborative quality improvement (CQI) through the Health Services Improvement Project (HSIP) to increase the coverage and quality of PHC for selected conditions, including hypertension. The investment focuses on 400 PHC facilities in eight districts in Khatlon and Sogd Oblasts. Through RBF, facilities and health care providers receive monetary incentives if they attain service coverage targets, including for the diagnosis and management of hypertension. About 200 clinics, through CQI, also adopted a system for monitoring and improving service delivery for hypertension and maternal and child healthcare, including 1) guideline-informed flowsheets to facilitate evidence-based diagnosis and treatment, 2) electronic medical records to monitor patient care, 3) monthly reviews of patient management and opportunities to improve services, and 4) data aggregation tools to assess facility-level trends [8].

Despite these strategies and investments since 2010, the 2017 DHS revealed that there are persistent gaps in the diagnosis and proactive treatment of hypertension in Tajikistan. Thus, in partnership with the MoHSP, the World Bank undertook a systematic assessment of service delivery for hypertension in PHC, embedded within the ongoing investments in RBF and CQI, to assess service delivery barriers and identify appropriate solutions to these challenges. This effort is an example of implementation research, which integrates systematic assessments within ongoing programs or policies to enable learning from real-world successes and failures. Through partnerships with local stakeholders, implementation research can ask the right questions, identify context-specific causes, and delineate feasible solutions, while drawing on the global evidence on what works in other contexts. A recently-published open-access report of the MoHSP and World Bank team summarizes the results of this assessment of service delivery of hypertension [9].

In this paper, we review the process of describing the cascade of care for hypertension and identifying potential solutions to address gaps in the continuum of care. The cascade of care framework has been used in a wide range of contexts to describe retention of service users in the continuum of care for conditions requiring multiple provider-user contacts, including HIV, tuberculosis, hypertension, and diabetes [10–14]. These studies provide useful descriptions of discontinuities in care use and correlations with patient characteristics. Our paper builds on these analyses by drawing on the cascade of care framework and using a mixed methods approach embedded in real-world programs to understand gaps in continuity of care and propose contextually appropriate solutions. Our paper may provide useful lessons for practitioners in similar contexts who aim to use the cascade of care to develop actionable insights for improving service delivery.

## Methods

We use mixed methods to identify drop offs in the hypertension cascade of care, to triangulate findings, and explore potential reasons and solutions for these gaps in care. Our quantitative approach includes analyzing household survey data and HSIP evaluation data to estimate retention across the care cascade. Our qualitative approach includes conducting focus group discussions with patients, providers, and health administrators and using thematic analysis to describe barriers and facilitators to hypertension care. We also conduct a scoping literature review of evidence-based approaches for improving health service delivery for hypertension and other chronic diseases. We integrate findings from this convergent mixed methods study design to recommend potential policy solutions to improve hypertension continuity of care in Tajikistan.

### Describing the hypertension care Cascade

**Fig. 1:**
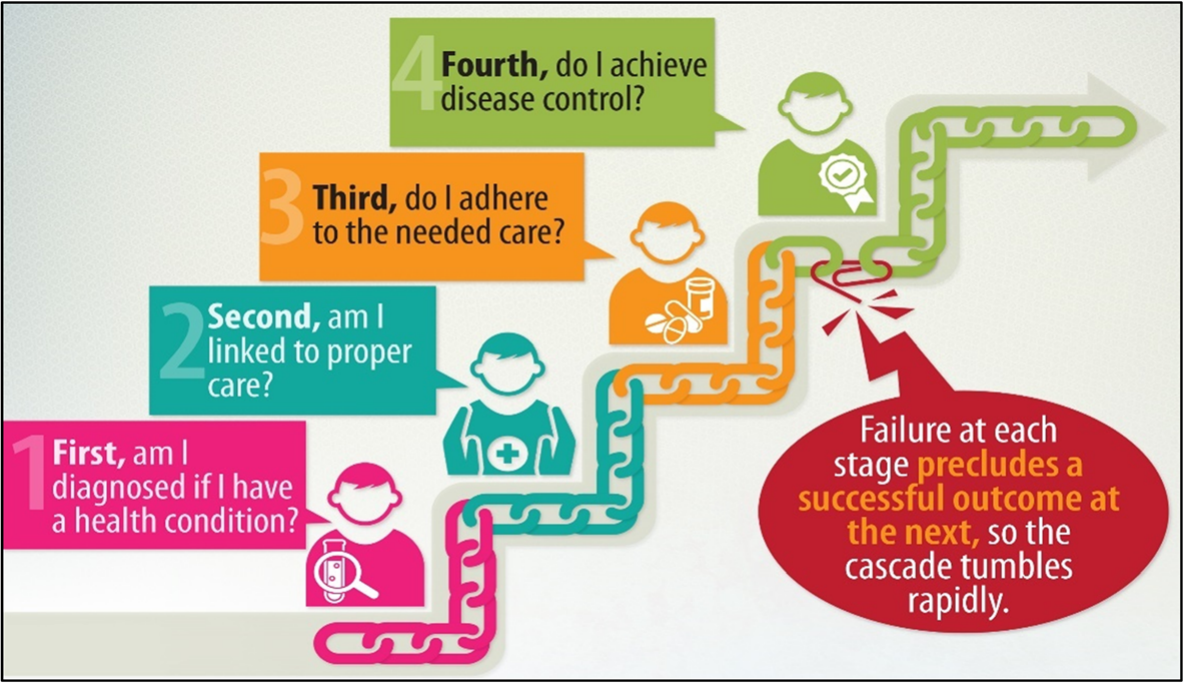
Overview of the cascade of care framework. Legend: The framework describes stages in the ideal progression from diagnosis of a chronic condition, through initiation and adherence to treatment, to disease control. Source: World Bank Delivery and Decision Science Global Solutions Group

The management of hypertension involves multiple contacts between the provider and service user—screening, diagnosis, treatment, and monitoring—to achieve and maintain BP control, which is essential to reducing complications and preventing mortality. The cascade of care disaggregates these contacts and provides a useful framework for examining patient progression and identifying drop-offs, that is a discontinuity of recommended care by service users along the cascade of care (Fig. 1).

**Fig. 2:**
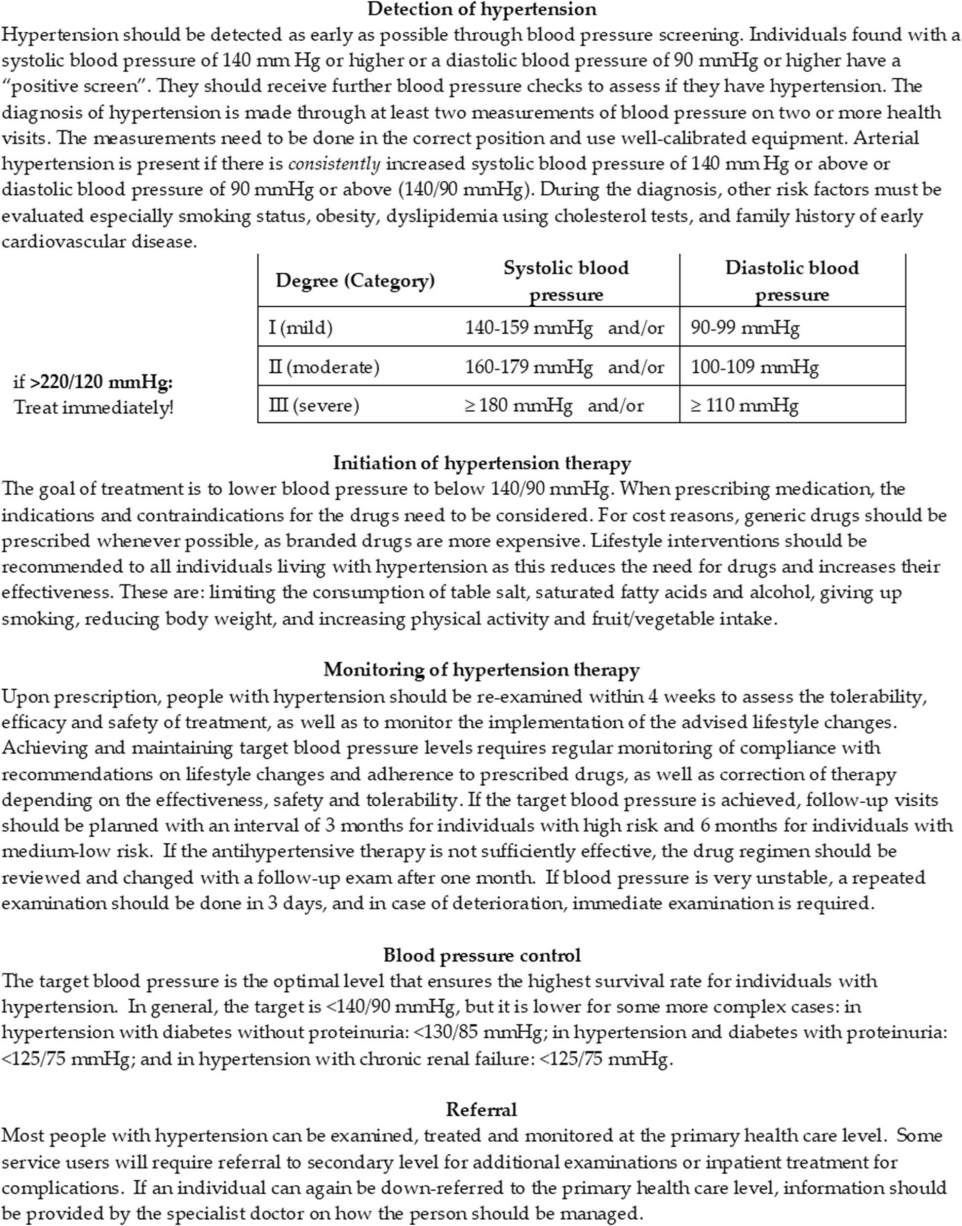
Summary of clinical guidelines for hypertension management in Tajikistan

We defined critical stages in patient-provider interactions from pre-diagnosis to the initial attainment of BP control and their accompanying measures:

1. *Diagnosis*: The proportion of people with hypertension in the catchment area of interest who are diagnosed at the facility level, where a diagnosis of hypertension follows at least two measurements of BP on two or more health visits with a systolic BP of 140 mmHg or higher or a diastolic BP of 90 mmHg or higher.
2. *Treatment Initiation:* The proportion of people diagnosed with hypertension who initiate treatment. To measure this, we defined the proportion who had been prescribed hypertension medication with a BP result on file.
3. *Treatment Monitoring:* The proportion of people who initiate treatment for hypertension, remain in care, and are followed-up by their service provider. A patient was considered being monitored by the care provider if there was evidence of BP being measured and recorded.
4. *Blood Pressure Control:* The proportion of hypertension patients who have achieved BP control. We defined control as BP < 140/90 mmHg or lower in complex cases, such as diabetes co-morbidity (Fig. 2).

We drew on existing data sources, including routine health information systems and household surveys conducted through the Demographic and Health Survey and a World Bank-funded Impact Evaluation. All quantitative data were available in unlinked anonymized format for analysis.

The World Bank 2018 Household Survey was conducted between April to July 2018 and measured the BP of all consenting adults over 18 years of age in households within the catchment areas of the facilities selected for investments in RBF and CQI in Sogd and Khatlon Oblasts. Eligible households either had a female member with a pregnancy in the past 2 years, or a family member above the age of 40 years. While these criteria imply that the survey may not have been representative of the adult population, it was the most recent objectively-measured rather than self-reported BP data within the facility catchment areas. In total, this survey provided data on 8443 adults in Khatlon and 3701 adults in Sogd Oblast, respectively.

The CQI database aggregated service user data from 198 facilities implementing CQI as part of the ongoing investment project, beginning in 2015. The catchment areas of these facilities included 237,501 individuals above 20 years in Khatlon Oblast and 78,518 individuals above 20 years in Sogd Oblast respectively. This patient-level database contained information on BP measurements, gender, and prescribed medication for hypertension following diagnosis.

The District Health Information System-2 (DHIS-2), the national health information system, provided population sizes for both Oblasts while the 2017 DHS included BP measurements and self-reported prescriptions for 10,718 women aged 15 to 49 years.

To estimate the number of hypertensive individuals in the CQI catchment areas, we multiplied the proportion of non-pregnant surveyed individuals with hypertension from the 2018 World Bank Household Survey (“estimated hypertension prevalence”) and the population in each catchment area. At the Oblast level, the proportion of hypertensive individuals was multiplied with the oblast population in the DHIS-2. Estimates for hypertension diagnosis, monitoring, and blood pressure control were limited to the CQI database and were unavailable for the broader population. The CQI database also documented for the registered hypertension cases the prescribed medicines, a diagnosis of diabetes, and body mass index. BP results within patient records were categorized by level of severity and by timing (ever/last 3 months).

By triangulating data sources, including household surveys and facility registries, we were able to estimate the proportion of hypertensive individuals who initiated care and were retained along each step of the care cascade. Graphical descriptions of the cascade of care in both Oblasts, by age group, were presented to facilitate assimilation by the target policy and practitioner audience. The quantitative data on retention along the cascade of care illustrated the magnitude of the burden of undiagnosed hypertension and the poor treatment outcomes among diagnosed cases, and motivated discussions on the causes of these gaps.

### Understanding the determinants of retention in hypertension care Cascade

To understand the context-specific reasons for drop-offs along the hypertension care cascade, we held focus group discussions (FGDs) with service users, health providers, and health care administrators in Khatlon and Sogd Oblasts. Service users included male and female adult patients from age 18 years and above, and pregnant women with a diagnosis of hypertension. We recruited health providers at the district level and health administrators who were heads of rural health centers (RHCs) or representatives of the Oblast Health Department. In total, we included 208 participants in 18 FGDs (Table 1).

**Table 1:** Characteristics of focus group discussion participants

*Participant Type*	*Oblast*	*CQI site type*	*Number of participants*	*Type of PHC*	*Location*
*Adult males in hypertension care*	Khatlon	Intervention	11	RHC	Balkhi
	Khatlon	Control	7	RHC	Jaihun
	Sogd	Intervention	12	RHC	Jabbor Rasulov
	Sogd	Control	11	RHC	Kanibadam
	Total		41		
*Adult non-pregnant females in hypertension care*	Khatlon	Intervention	11	RHC	Balkhi
	Khatlon	Control	12	RHC	Jaihun
	Sogd	Intervention	12	RHC	Jabbor Rasulov
	Sogd	Control	12	RHC	Kanibadam
	Total		47		
*Pregnant women in hypertension care*	Khatlon	Intervention	12	DHC	Balkhi
	Khatlon	Control	12	DHC	Jaihun
	Sogd	Intervention	12	DHC	Jabbor Rasulov
	Sogd	Control	12	DHC	Kanibadam
	Total		48		
*Health care providers*	Khatlon	Intervention	12		Balkhi
	Khatlon	Control	12		Jaihun
	Sogd	Intervention	12		Jabbor Rasulov
	Sogd	Control	12		Kanibadam
	Total		48		
*Heads of Oblast health departments*	Khatlon		12		Bokhtar
	Sogd		12		Khujand
	Total		24		

*RHC* Rural Health Centre, *DHC* District Health Centre

The FGD guides were developed collaboratively by technical experts within the World Bank and Tajikistan. World Bank experts in service delivery, implementation research, and clinical medicine drafted discussion guides with probes to identify the causes of drop-off and retention at each stage in the care cascade, which were reviewed and refined for wording by local experts involved in hypertension care. The discussion guides were also reviewed by local stakeholders involved in the implementation of the ongoing investment project for completeness and by other facilitators of the FGD to ensure that the translated guides were worded to be consistent with the desired meaning.

The final FGD guides were organized by stage in the care cascade and concluded with an invitation to discuss how high BP could be avoided, that is primary prevention measures. See Additional files 1, 2, 3 for full guides. For patients with hypertension, the questions in the discussion guide nudged patients to reflect on the positive and negative experiences with diagnosis and treatment, and the potential reasons for patients to discontinue care. Provider and health administrator discussion guides were structured similarly, to describe the current state of hypertension care, including challenges, their causes, and suggestions for improvement. Health administrators were also asked to describe oblast-level factors relating to the care cascade, such as programmatic support for diagnosis, treatment initiation, and follow-up, and to reflect over potential interventions to address causes of drop-offs in the care cascade.

Leveraging established working relationships with RHC staff under the HSIP, the FGDs with patients were carried out in RHC facilities. Patients that met the eligibility criteria on age (18 years or older), hypertension status, and pregnancy status were recruited by RHC family doctors and nurses and invited to participate in a discussion at their RHC. Providers and administrators were contacted directly by HSIP staff and encouraged to participate in a discussion at their Oblast health department or district health center.

Each FGD was led by two to three individuals, some of whom were HSIP staff, one of whom served as a facilitator while the other served as note-taker. The purpose of the FGD was discussed, and participants provided written consent to be involved in the audio-recorded exchange. FGDs were conducted over 6 days in November 2018, overlapping with supervision visits by HSIP staff as part of the investment project, with each FGD lasting about two hours and including up to 12 participants.

Following the FGDs, detailed summaries were created, drawing on the audio recordings and notes from each session and translated into English for analysis. Two researchers independently conducted a thematic analysis of each summary to identify the barriers and facilitators of retention in the hypertension care cascade. Differences in themes were discussed and reconciled, and the final list of themes was reviewed with a member of the local team that led the FGDs to ensure consistency with initial findings. The final analysis of FGDs described barriers and facilitators by cascade stage and included patient-, provider-, and administrator-level perspectives, which are fully described in a recently-published report [9].

### Identifying fit-for-purpose solutions to improve retention in the hypertension care Cascade

We defined fit-for-purpose solutions as interventions that met three criteria: 1) focuses on a barrier to or facilitator of retention in hypertension care identified in the diagnostic process; 2) empirical evidence of effectiveness in the published or grey literature, and 3) perceived by local stakeholders as applicable to PHC in Tajikistan.

Based on the identified barriers to and facilitators of retention in hypertension care, the characteristics of appropriate solutions were defined as illustrated in Table 2 below:

**Table 2:** Characteristics of main barriers and appropriate solutions for hypertension care

Barrier	Solution
Wrong understandings of disease and its therapy among people living with the illness	Enable understanding of hypertension and its therapy among people with hypertension
The high time and monetary costs of seeking care	Reduce the time and monetary costs of seeking care
Ambiguous and inappropriate clinical guidelines, particularly for remote under-resourced areas	Improve the ease of understanding of clinical guidelines and adapting them to ensure their relevance to the context
Shortages of human resource for health and equipment for blood pressure monitoring	Ensure the supply of trained and equipped health workers
A lack of support from peers, family, providers, and the community for initiation and adherence to hypertension care	Incorporate support from peers, families, and communities

**Fig. 3:**
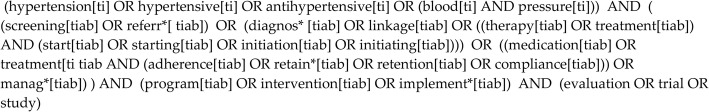
Literature search terms for scoping review

In October 2018, we conducted a review of interventions aimed at addressing the identified barriers in the hypertension care cascade. Using PubMed, we searched for English or English-translated articles published between 2000 and 2018 that evaluated an intervention’s impact on a care cascade-related metric, such as percent referred to care, percent adherent to medication, and change in BP (Fig. 3).

We screened the abstracts of resulting articles and retained those that met our inclusion criteria of evaluating a program or intervention’s measurable hypertension care cascade-related outcome. We extracted the following information into a spreadsheet template: the country the intervention took place in, the target population, care cascade focus area, type of program or policy, health service level, health personnel involved, sample size, primary and secondary outcomes, and any available cost information. Whether interventions resulted in statistically significant improvements in outcomes was also indicated as a measure of empirical effectiveness. Studies were also added to our review if conversations with local providers and administrators suggested further research into specific intervention types, including abbreviated provider care guides.

Of the 150 included studies, 14 (9.3%) occurred in the World Bank-defined region of East Asia and the Pacific, 22 (14.7%) in Europe and Central Asia, 10 (6.7%) in Latin America and the Caribbean, 2 (1.3%) in the Middle East and North Africa, 67 (45.3%) in North America, 10 (6.7%) in South Asia, and 21 (14%) in Sub-Saharan Africa. A third of the studies (*n* = 49) focused on community-based interventions across all hypertension cascade stages, while two-thirds (*n* = 108) of studies focused on primary or secondary care interventions during the treatment compliance stage alone. In some cases, the studies targeted more than one health service level or cascade stage.

**Fig. 4:**
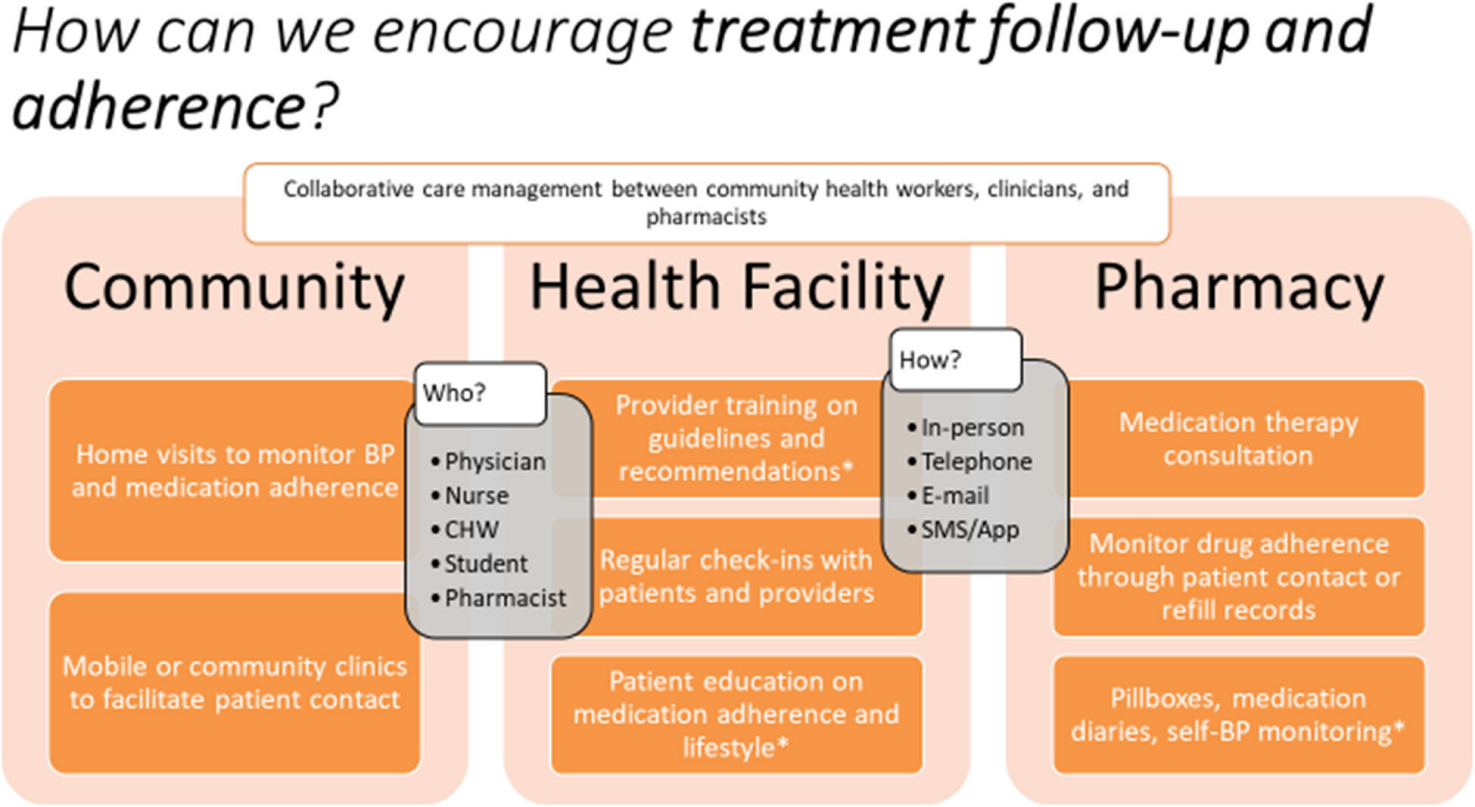
Discussion prompt on interventions to improve treatment follow-up and adherence. Legend: CHW: Community Health Worker; BP: Blood Pressure

Our literature review aimed to scope the range of clinical and non-clinical interventions that addressed the identified barriers to retention in the hypertension care cascade and aligned with the characteristics of appropriate solutions to these barriers. The literature review served as a practical tool in guiding discussions on possible policies or programs for hypertension care in the Tajikistan context. Thus, interventions found to be effective in other contexts were summarized by intervention scope, setting, and personnel, and presented to policymakers at the MoHSP and during FGDs with health administrators, providers, and patients (Fig. 4). During this discussion, the initial list of proposed interventions was narrowed to a final set of fit-for-purpose solutions based on stakeholder feedback on the perceived feasibility of implementing the interventions in the Tajikistan context.

## Results

Detection of hypertension and facility registration are necessary steps towards the initiation of appropriate management in Tajikistan. To illustrate the utility of this implementation research methodology for providing actionable insights, we describe our results of diagnosis of hypertension, the first step of the cascade, below.

About one in three adults in Khatlon and Sogd Oblasts are hypertensive. Hypertension is even more common in adults above 65 years of age, with an estimated prevalence of 62% in Khatlon and 68% in Sogd Oblast, respectively, in this age group (*p* = 0.012). There are also statistically significant differences across gender, with a higher proportion of males (36%) hypertensive compared to females (31%, *p* < 0.001) in both Oblasts combined. Moderate to severe hypertension was relatively more common in older versus younger cases, and in cases in Khatlon Oblast compared to Sogd Oblast.

As shown in Table 3, of the estimated half a million people with hypertension in either Oblast, about 10% have been diagnosed and registered in a facility in Khatlon Oblast, while only about 5% have been diagnosed and enrolled in a facility in Sogd Oblast. These differences in diagnosis rates were partially a reflection of differential screening rates. Between 2016 and 2017, there was a 43% increase in new hypertension cases diagnosed in Khatlon, relative to a 5% increase in Sogd, where diagnosis rates fell among people aged 65 years and above. While 52% of males were screened for hypertension in Khatlon in 2018, only 43% were screened in Sogd.

**Table 3:** Estimated number of hypertensive persons and hypertension cases diagnosed in the Oblasts

	Khatlon			Sogd		
	Estimated prevalence	Estimated number hypertensive^a^	Number diagnosed (%)	Estimated prevalence	Estimated number hypertensive	Number diagnosed (%)
OBLAST						
Males ≥20 yrs	35%	266,000	19,968 (7.5%)	36%	262,000	10,156 (3.9%)
Females ≥20 yrs	30%	232,000	31,292 (13.5%)	34%	293,000	15,401 (5.3%)
Total ≥ 20 yrs	33%	498,000	51,260 (10.3%)	35%	555,000	25,557 (4.6%)

Sources: World Bank 2018 Household survey, DHIS2 for Oblast population numbers and registered as hypertensive in 2017

^a^ Hypertension = Elevated blood pressure (SBP ≥140 mmHg or DBP ≥90 mmHg)

Discussions with key stakeholders revealed that screening and diagnosis rates are negatively affected by a lack of understanding of hypertension in the general population; ambiguous clinical protocols; the low priority given to hypertension screening; and the limited capacity to reach rural and remote communities. Prenatal care and community screening were identified as avenues that have been successful at identifying undiagnosed cases. We review these themes below.

It is common for hypertensive individuals to be unaware of their condition and for the first encounter with the health facility to occur following complications of chronic hypertension. The connection between high BP and these complications, including cardiovascular diseases, is also not well understood by individuals with hypertension.

*“Patients do not come for screening because they don’t know the risks and negative consequences of hypertension.” (Participant 5, Health Administrator, Khatlon Oblast).*

Even in the absence of information barriers, communities face costs in terms of money and time to obtaining a screen for hypertension in the health facility or at home. Households in remote areas are often also located a far distance from the nearest health center and may find the facility challenging to reach. These locations are also less likely to participate in community screening. Among rural populations that may not be remote, farm work during the day may prevent individuals from being screened by healthcare workers, even at home.

On the supply-side, particularly in rural and remote areas, an insufficient supply of equipment and human resources has limited the ability of service providers to screen their catchment population for hypertension, at home or in the facility. Sphygmomanometers are not replaced and calibrated regularly, reducing their accuracy in measuring BP. A high caseload for limited health workers implies that routine screening in the community and facility is de-prioritized. Furthermore, current protocols have an unclear scope of work for each health care level, lack clear guidance for service delivery in remote and rural areas, provide insufficient information on case finding, treatment adherence and common barriers to adherence, and do not consider the perspectives of providers and service user. There are also no readily-available guides that providers could refer to when needed.

*“Clinical protocols are available for the medical workers, but the language in the protocols are not easy to understand, and not adapted to existing conditions. When national protocols are being developed, they should consider realistic conditions in rural areas and geographic landscapes.” (Participant 4, Health Care Provider, Sogd Oblast).*

In terms of policy and programming, health administrators were unaware of the high hypertension burden and the levels of under-diagnosis. As analysis related to the burden of hypertension, diagnosis, and management of cases, was not routinely done, these statistics could not inform strategic plans for the health sector. In addition to constraints from limited financing, the NCD strategy, which is the guiding policy document, does not include specific recommendations to improve service delivery for hypertension.

*“The Chief cardiologist collects data on hypertension from the districts. However, we do not analyze the data on hypertension closely. I think we should.” (Participant 7, Health Administrator, Sogd Oblast).*

Antenatal care and community screening initiatives are effective at facilitating early detection of hypertension but are not regularly conducted or targeted at underserved areas. Specifically, all pregnant women currently undergo BP checks during routine antenatal care visits, regardless of age. Through the “caravan of health,” an outreach campaign in which health care providers visit remote areas for one-time health consultations, screening for hypertension has been provided by the Ministry of Health and Social Protection, and Provincial Health Administrators.

Following the literature review and discussions with key stakeholders on the feasibility of interventions to address the above challenges, we identified fit-for-purpose solutions to increase hypertension diagnosis in Tajikistan. These solutions included mobilizing faith-based organizations, scaling up screening through May Measurement Month and health caravans, leveraging service user interactions with pharmacy care, introducing job aids for providers, and task-shifting to increase provider supply. We review these solutions below.

Faith-based organizations have a unique influence on the population, and religious leaders can champion a wide variety of social causes. This influence can be leveraged to promote awareness of hypertension and to facilitate early diagnosis, for instance, by locating screening activities around mosques and religious events. For example, case detection of tuberculosis in Asia has also been carried out by screening people in mosques [15].

May Measurement Month and health caravans are both models that bring diagnostic opportunities closer to the community, reducing geographic and financial barriers to health care access and entry to care. These interventions are particularly relevant for people in informal employment, such as farming, with working hours that overlap with the times the facility is open. May Measurement Month is a global campaign initiated by the International Society of Hypertension that encourages blood pressure screening on and around World Hypertension Day (May 17) [16]. World Hypertension Day has been observed in over 90 countries, testing over a million people in a single year, and raising awareness of the importance of BP monitoring and therapy for hypertension [17]. Furthermore, while health caravans have been used sporadically for outreach since 2009 in Tajikistan, a systematic approach to deploying them, that focuses on rural and remote areas, could facilitate broader awareness of hypertension, screening for early diagnosis, and follow-up of care within the community. In South Africa, a similar model of community screening led to 50% of individuals being linked to care within a month [18].

Given the shortage of skilled health care staff to provide hypertension care, models that transfer simple tasks related to hypertension care to other cadres can expand the human resource pool very quickly in the short term. In this vein, practicing nurses, retired nurses, medical students, and nurses-in-training can undertake some tasks in hypertension management. A similar model has been used in Singapore, where medical student volunteers screened low-income households, made referrals for hypertensive individuals, and conducted follow up, leading to over 50% of untreated hypertensives seeking treatment [19].

Finally, introducing easy-to-use job aids can address concerns among providers about the relevance, accessibility, and clarity of guidelines for care. Job aids can provide practical and evidence-based guidance to health workers that are easy to refer to even during interactions with service users. In combination with mobilizing lower skilled health workers for hypertension screening and care management, using simplified job aids may be necessary to promote high-quality care across all skill levels. In a multi-component hypertension intervention in Bangladesh, Pakistan, and Sri Lanka, checklists were used by community health workers, nurses, and general practitioners in screening and managing care for hypertensive individuals, and resulted in substantial BP reductions [20].

While the ongoing investments in monetary incentives for health care providers and mechanisms for facility quality improvement have facilitated increases in the diagnosis of hypertension, our study revealed that there were other barriers to care that prevented case detection. A series of in-depth conversations with stakeholders and reviews of the global literature suggests that these gaps in hypertension diagnosis can be addressed in part by scaling up community screening, mobilizing lower-skilled health care providers and pharmacies, and providing accessible and relevant guides for health workers.

Through the ongoing investment project, there is an entry point to introduce these solutions within the 400 participating PHCs in Sogd and Khatlon Oblasts, to monitor the resulting changes in hypertension detection rates, and to engage iteratively in the above process of diagnosing other gaps in service delivery.

## Discussion

Our study is an example of implementation research that is motivated by and embedded in larger health system programs and policies. In this case, implementing the HSIP, which targeted health facility performance at the PHC level, revealed the need to better understand the current state of hypertension care in the population. We leveraged HSIP evaluation data to illustrate the magnitude of undiagnosed hypertension and the extent to which diagnosed individuals engage in care. We add to and complement HSIP efforts, highlighting opportunities and tailored solutions (e.g. screening, task shifting, and job aids) that aim to capture and retain more hypertensive individuals in care. Independent of the health system’s long-term adoption of HSIP initiatives, our research emphasizes the need to address persistent challenges in identifying hypertensive individuals and connecting them to health services at every stage in the cascade of care.

The cascade of care framework provided a systematic way of assessing the state of hypertension care within PHC settings in Tajikistan. It confirmed that existing strategies and programs might need to be reinforced with interventions that identify undiagnosed hypertension cases while addressing gaps in other stages of the cascade. The series of in-depth discussions with local stakeholders that followed illustrated the multi-level factors that were perceived to influence drop-offs along the cascade of care and provided guidance on the interventions that were best able to target the identified factors. Scoping the literature provided an array of options to address the causes of drop-offs. These three activities – quantitatively describing the cascade, in-depth discussions, and the literature review— informed the final list of intervention options for policymakers that were tailored to the Tajikistan context.

Below, we reflect on lessons from this implementation research effort that can inform similar studies.

### Collaboration with local stakeholders

Implementation research in health systems draws on a range of contributors, including researchers, national policymakers, local administrators, program managers, health care providers, and service users. Local stakeholder involvement was critical to the design and conduct of this implementation research project, increasing its potential to directly and quickly translate into policy and program implementation. The HSIP, through which RBF and CQI are being implemented to improve care for hypertension, is the result of direct collaboration between the World Bank and the Tajikistan MoHSP. Monitoring data collected through the HSIP, and subsequent discussions between the World Bank team and the MoHSP, highlighted the persistent gaps in diagnosis and management of hypertension cases.

Furthermore, the implementation of HSIP involves close collaboration with health administrators at the Oblast level. Thus, when the need to assess the current state of hypertension care was identified, ongoing and active partnerships were available to leverage for the new scope of work. In describing the cascade of care, the MoHSP and other local stakeholders advised the team on available data sources and participated in collecting the required information. Following the literature review, local stakeholders validated the initial findings and identified feasible interventions. Most crucially, for the FGDs, existing partnerships with rural health centers allowed for swift recruitment of participants and coordination of data collection at the rural health centers. High-level policy discussions with the MoHSP during implementation support visits for the Tajikistan HSIP served as an avenue to provide updates on activities within the implementation research effort and receive guidance on which areas to focus. Finally, in collaboration with MoHSP, the team conducted observations of hypertension service delivery in Yavan district of Khatlon Oblast to corroborate initial findings on drop-offs from the cascade of care and their causes. The collaborative nature of the study generated demand for the results which were presented at the Asian and Commonwealth of Independent States Congress of Cardiologists and Therapeutics Conference in Dushanbe in April 2019.

### Triangulating data sources to describe and understand service delivery patterns

To understand patterns of patient progression along the cascade of care, we drew on quantitative and qualitative data sources. Our quantitative data was sourced from program implementation and evaluation activities that focused on catchment areas of rural health centers Khatlon and Sogd oblasts. Estimating the prevalence of hypertension, its diagnosis, and management using multiple data sources yielded estimates that approximated those from national-level representative surveys. For example, our estimates of the prevalence of hypertension (35% in males and 30% in females) were very similar to those found in the Tajikistan 2016 STEPS survey for chronic disease risk factor surveillance (32.3% on average, with higher values in males than females) [21]. Thus, we are reasonably confident in the salience of our main finding of the low proportion of hypertensive individuals that are connected to and remain in care.

The availability of data constrained the description of the cascade of care. For example, while we were able to document if any medication was prescribed, there was no data on whether lifestyle changes were recommended. Such changes to physical activity and diet, or smoking and alcohol consumption, are essential to hypertension management and are known challenges to blood pressure control [22]. While our focus group discussions revealed that some patients were advised to reduce salt intake and increase physical activity, future research might examine the extent of (and adherence to) non-pharmacological treatment at the PHC level and explore the possibility of lifestyle modification education delivered by lower skilled workers. Furthermore, the absence of data on risk categorization, such as lipid profiles and co-morbidity, and on self-monitoring, prevented the team from further describing treatment adherence and follow-up by risk category, and BP monitoring at home. Nonetheless, combining data sources using reasonable assumptions enabled us to derive the striking quantitative and graphical descriptions of the cascade of care, which have motivated ongoing discussions on strengthening hypertension care in Tajikistan and pointed attention to a hitherto neglected aspect of the cascade, that is undiagnosed cases of hypertension.

The qualitative data collected through FGDs revealed, in several cases, causes of drop-offs and opportunities for intervention that were unique to Tajikistan. For example, there were several discussions about wrong beliefs about hypertension and its management in this context, including the fear of potential addiction to hypertension medication, stigma facing young women who were diagnosed with chronic conditions, and the assumption that individuals younger than 40 years did not require screening for hypertension. In reflecting over potential solutions to the service delivery challenges, the “Chaikhana” (tea house) meetings, which are social gathering places specific to the Central Asian context were identified as a means of engaging men in discussions on hypertension and its treatment. Furthermore, health caravans, which have been used since 2009 for improving health care access in rural and remote areas in Tajikistan, was highlighted as an avenue for closing gaps in screening for hypertension in these localities.

### Fit-for-purpose solutions as the intersection of feasibility and effectiveness

It was essential, in defining a solution to a service delivery challenge, that the intervention did not just have evidence of effectiveness, but that it could be implemented within the Tajikistan context. This required discussions on the intervention components, their administrative and clinical human resource needs, cost, cultural acceptability, and the broader policy and financing environment. Combining the literature review with the FGDs enabled us to pass the array of options identified from other contexts through the filter of the judgment of patients, providers, and administrators on the applicability of these interventions to Tajikistan. In some cases, interventions that were empirically effective were not feasible. For example, while the literature showed that expansion of pharmacist and nursing roles could allow for closer treatment monitoring, there are legal limitations to who can sell prescription medications and make clinical decisions in Tajikistan. Also, there were interventions that were not identified in the literature review but were highlighted by policymakers as applicable to the Tajikistan context. Thus, the final decision on the list of fit-for-purpose solutions was arrived at through deliberating with local stakeholders.

## Conclusions

The burden of cardiovascular diseases is growing in Tajikistan, and hypertension is a key risk factor. A partnership between the World Bank and the Ministry of Health and Social Protection of Tajikistan, thus, aimed to improve the quality of hypertension care through RBF and CQI within primary care. However, project monitoring data and a household survey in 2017 revealed there were persistent gaps in the diagnosis and management of hypertension. This finding motivated a collaborative effort to describe patient progression in hypertension care and its determinants. Our experience undertaking this effort highlights the necessity for close partnerships with local stakeholders in designing and conducting similar studies, the utility of triangulating routine and survey data to understand service delivery patterns, and the importance of the intersection between feasibility and effectiveness in defining fit-for-purpose solutions to service delivery challenges.

## Supplementary information

**Additional file 1.** Focus Group Discussion Guide for Hypertension Patients (Pregnant and Non-pregnant). A document describing eligibility for participation, instructions for the interviewer, and discussion prompts.

**Additional file 2.** Focus Group Discussion Guide for Hypertension Care (Administrators). Description: A document describing eligibility for participation, instructions for the interviewer, and discussion prompts.

**Additional file 3.** Focus Group Discussion Guide for Hypertension Care (Providers). A document describing eligibility for participation, instructions for the interviewer, and discussion prompts.

## Data Availability

The datasets used and/or analyzed during the current study are available from the Ministry of Health and Social Protection on reasonable request, which includes submission of contact information, a description of the proposed analysis, and specification of data type requested to study authors. Qualitative data may not be available in full due to personally identifiable information.

## Abbreviations

CQI: Collaborative Quality Improvement
DHC: District Health Centre
DHIS-2: District Health Information System 2
DHS: Demographic and Health Survey
FGD: Focus Group Discussion
GDP: Gross Domestic Product
NCD: Non-Communicable Diseases
PHC: Primary Health Care
RCMSI: Republican Centre for Medical Statistics and Information
RHC: Rural Health Centre
WHO: World Health Organization
