# Supplementary material for: Challenges and opportunities in the continuity of care for hypertension: a mixed-methods study embedded in a primary health care intervention in Tajikistan
Source: BMC Health Serv Res. 2019 Dec 3;19:925. doi: 10.1186/s12913-019-4779-5 (PMC6889695; doi:10.1186/s12913-019-4779-5)
Supplement: Supplementary file 1 — Additional file 1. Focus Group Discussion Guide for Hypertension Patients (Pregnant and Non-pregnant). A document describing eligibility for participation, instructions for the interviewer, and discussion prompts. [file 12913_2019_4779_MOESM1_ESM.docx]

**Hypertension Control Cascades**

Focus Group Discussion Guide for Hypertension Patients (pregnant and non-pregnant)

Target Audiences: FGDs to be conducted in **health facilities** associated with the selected **study villages**

Eligibility: Male, pregnant female hypertension patients & non-pregnant female hypertension patients aged 18 years or above. Aim for pariticpants from these sub-categories: HTN patients stable on treatment and meeting BP target; HTN patients “unstable” on treatment, repeatedly failing BP target; HTN patients newly on treatment (e.g. first 3 months), and long-term HTN patients.

**Instructions for the Interviewer**

**Step 1: Informed Consent**

*Ask each participant for about XXXX of their time. Introduce yourself and the study. Begin the informed consent as per the training. Leave the informed consent sheet with each participant. If consent is granted for FGD participation* ***and*** *audio-recording, proceed to next step*

**Step 2***:* **Identification of consented participant**

*Complete the basic demographic table for each consented participant as they sign in.* *This form will have an ID number for each participant. Make sure that the note taker has the correct ID numbers recorded on his/her notes prior to beginning and that participants have their correct “Letter Label” hanging around their neck to help the note taker with identification of participants.*

**Was written informed consent obtained for each participant before the start of the FGD (including permission by each to audio record this discussion)?**

**YES ________ (proceed with FGD)**

**NO** ________ (**STOP and only continue with the individuals who have consented to participate)**

**Step 3***:* **Introduction**

*Read the following statement. Please repeat the statement translated into the local language based on primary language used by the group*

“Thank you for agreeing to participate in this discussion. My name is _______________. I will be asking you the questions. My partner _______________ will be taking notes on the things you have to say.

We want to understand your views on hypertension care available at your local health facility and in your community. To make these better, it is important that we understand what you as patients know about these services and how you are experiencing them. Our discussion will cover the stages patients go through: **Testing for blood pressure, starting treatment and taking treatment**. Please feel free to tell us whatever you are comfortable sharing. You should also remember that you do not have to share anything that you are not comfortable sharing. We will not write down your name. There are no right or wrong answers, so please be honest and tell us what is true for you and your community. If at any point during the discussion, you decide to leave, you are free to do so. This will also not incur any penalty. Are you ready to begin?”

**Step 4:** *Complete the fields below and then start with the FGD. Start by reiterating the importance of confidentiality within the group. As you ask the questions, please probe appropriately to gain as much depth on the topics as possible.*

**Facilitator name** ____________________ **Note-taker name ________________**

**Date (dd/mm/yyyy)** ____/ ____/ 20___

**Facility ___________________________**

**Time Start** _____: ____ **Time Finish** _____: _____

**Supervisor name ___________________________**

**1 Diagnosis (pre-treatment phase)**

- 1. Do you think the diagnosis of hypertension is well done at your health facilities?

***First*** *allow patients to respond.* ***Then probe for:*:**

- Positive aspects of being diagnosed with hypertension? (most important three positives)
- Negative aspects of being diagnosed with hypertension? (most important three negatives)
- After one high blood pressure measurement, when were you re-measured to see if there is hypertension?
- What could put people off from having their blood pressure checked?
- Perceptions of efficiency, friendliness, confidentiality of diagnostic process
- Views on health information provided at diagnosis
- If you have questions at hypertension diagnosis, what is available to you? (e.g. resources, materials, staff, contact numbers)
- Comments on the roles of the different health staff involved in diagnosis?
  1. How could hypertension diagnosis be improved?

***First*** *allow patients to respond.* ***Then probe for:***

- Did the health information you received at diagnosis meet your needs?
- What services would help people get their blood pressure checked?
- What concrete improvements could be made in the service offer for hypertension diagnosis? (seek specific detail on e.g. counselling content, referral) *(Write these down on a flipchart)*

1. **Treatment start (initiation)**
   1. What are your views on how treatment for high blood pressure is started by your doctor or nurse?

***First*** *allow patients to respond.* ***Then probe for:***

- What was positive during treatment initiation? (most important three positives)
- What was negative or unpleasant during treatment initiation? (most important three negatives)
- What could prevent people from starting hypertension treatment?
- How quickly were you offered hypertension treatment? Was it a prescription or actual drugs? By same doctor/nurse as the one who told you that you had high blood pressure? Same place? How simple was the process of starting treatment? [pre-treatment loss]
- Were there any treatment options presented (such as: drugs only vs. drugs in combination with life style changes/weight loss program; fixed-dose combination vs. multiple individual drugs)
- Do some people start treatment and stop very quickly? Why?
- Perceptions of efficiency, friendliness, patient choice at treatment start
- Views on the information, education, communication (IEC) provided at treatment start? What topics are covered?
- If you have questions on hypertension treatment, what is available to you? (e.g. resources, materials, staff, contact numbers)
- Comments on polypharmacy (dug labelling, clarity of regimens, ease of prescriptions/refills)
- Comments on the roles of the different health staff involved in treatment start?
  1. How could the starting of hypertension treatment be improved?

***First*** *allow patients to respond.* ***Then probe for:***

- Does the IEC at treatment start meet the needs of *individuals*?
- How can the health service help new hypertension patients with getting the treatment started instead of being left without treatment and care?
- What concrete improvements could be made at the stage of starting hypertension treatment? (seek specific detail on e.g. IEC on drug’s adverse effects, drug refill, fixed dose combinations, etc.) *(Write these down on a flipchart)*

1. **Treatment maintenance and monitoring**
   1. What do you think about the long-term care for hypertension patients by your health facilities?

***First*** *allow patients to respond.* ***Then probe for:***

- What works well for patients in long-term hypertension care?
- What are important challenges for patients in long-term hypertension care?
- What are the reasons patients stop taking their hypertension drugs?
- Once on treatment, do healthcare workers support patients in specific ways to help with sticking to the treatment? What services are offered? Do they ever call? Give education? Ask you/remind about your medication?
- Adherence to non-pharma treatment (healthy eating, weight loss, physical activity, smoking cessation)?
- What are the views on clinic visit schedules and monitoring tests? [could probe on opening times, queues for chronic patients, getting an appointment, convenience of clinics for consultations, lab tests, lab results, drug refill]
- Do patients know when treatment is working well? (communication of reaching/failing BP target, but also meeting targets cholesterol, weight/BMI, glucose)
- Perceptions of efficiency and convenience of long-term hypertension care? [is it a one-stop-shop]
- Comments on the roles of the different health staff involved in long-term care?
  1. Can you tell me about the strategies that you or some of the people in your community use to help make sure they take their hypertension treatment every day as prescribed? *(Write these down on a flipchart)*

***First*** *allow patients to respond.* ***Then probe for:***

- Personal/family strategies (e.g. alarm clocks, reminders, pill boxes, family support)
- Community supported strategies (e.g. clubs, support groups, specific NGO services)
  1. What do health facilities do to promote adherence among their hypertension patients and help them stay in care?

***First*** *allow patients to respond.* ***Then probe for:***

- Facility supported strategies (e.g. Buddy system, text reminders, alternative places to pick up meds, longer prescriptions, triage into chronic track, counselling, timing of counselling, tracing)
- System strategies like integration (one-stop-shop drug), subsidies/co-payment, insurance, benefits
  1. What do health facilities do to specifically support patients who fail their BP target while on treatment?

***First*** *allow patients to respond.* ***Then probe for:***

- Increased BP monitoring intervals?
- Diagnosis of root-cause of non-adherence? Specific counselling/IEC?
- Treatment step-up?
- Specific staff training/focal point?
- What else would work for these patients who struggle to reach BP target?
  1. How could long-term hypertension care be improved?

***First*** *allow patients to respond.* ***Then probe for:***

- Is the care provided sufficiently patient-centered/individualized?
- Are test results used and communicated as part of patient care?
- What could health facilities do differently to promote adherence?
- Are there changes needed to make these work well in the community? What? Why?
- Do different groups need different support?
- What else is needed? What other support would help?

*(Write these down on a flipchart)*

- 1. (Among pregnant patients) How can care for hypertension in pregnancy be maintained post-pregnancy?

***First*** *allow patients to respond.* ***Then probe for:***

- What challenges prevent women from continuing treatment for hypertension after pregnancy, where necessary?
- Is there any support from the health facility or provider for women who started treatment for hypertension during pregnancy, after delivery? If yes, describe support and roles of different providers.
- What other services can improve transition of hypertension care after pregnancy?

1. **Primary prevention**
   1. How can Tajikistan prevent people from having high BP in the first place?

***First*** *allow patients to respond.* ***Then probe for:***

- Individual/family strategies?
- Health services strategies?
- Community strategies?
- Which strategies will work best for adults? For children?
- Is stigma (of obesity or of diabetes) an issue? What is needed to address stigma?
- If prevention strategies are not working, why not?
- What would makes them more effective?

*(Write these down on a flipchart)*

***Thank the participants for their time and contribution. Remind them that if any of them wish to share anything in private they can come to talk with you separately.***

***Close the meeting and complete the time the discussion finished on the first page.***

***Ask the supervisor to check consent papers, the demographic table, the notes and the auto-recording, and to sign on the first page. The supervisor is responsible for safekeeping of these items, once handed over.***
