# Supplementary material for: Challenges and opportunities in the continuity of care for hypertension: a mixed-methods study embedded in a primary health care intervention in Tajikistan
Source: BMC Health Serv Res. 2019 Dec 3;19:925. doi: 10.1186/s12913-019-4779-5 (PMC6889695; doi:10.1186/s12913-019-4779-5)
Supplement: Supplementary file 2 — Additional file 2. Focus Group Discussion Guide for Hypertension Care (Administrators). Description: A document describing eligibility for participation, instructions for the interviewer, and discussion prompts. [file 12913_2019_4779_MOESM2_ESM.docx]

**Hypertension Control Cascades**

Focus Group Discussion Guide for Hypertension Care (Administrators)

Target Audience: FGDs to be conducted with **oblast level administrators involved in hypertension care programs or planning in** the selected **study villages**

Eligibility: Oblast-level administrators involved in hypertension care programs or planning in government health facilities including ANC services

**Instructions for the Interviewer**

**Step 1: Informed Consent**

*Ask each participant for about half an hour of their time. Introduce yourself and the study. Begin the informed consent as per the training. Leave the informed consent sheet with each participant. If consent is granted for FGD participation* ***and*** *audio-recording, proceed to next step*

**Step 2***:* **Identification of consented participant (continue from here on a set date, afternoon hours in a clinic, allowing for 2 hours of discussion)**

*Complete the basic demographic table for each consented participant as they sign in.* *This form will have an ID number for each participant. Make sure that the note taker has the correct ID numbers recorded on his/her notes prior to beginning and that participants have their correct “Letter Label” hanging around their neck to help the note taker with identification of participants.*

**Was written informed consent obtained for each participant before the start of the FGD (including permission by each to audio record this discussion)?**

**YES ________ (proceed with FGD)**

**NO** ________ (**STOP and only continue with the individuals who have consented to participate)**

**Step 3***:* **Introduction**

*Read the following statement:*

“Thank you for agreeing to participate in this discussion. My name is _______________. I will be asking you the questions. My partner _______________ will be taking notes on the things you have to say.

We want to understand your views on hypertension care provided by the public health sector. Our discussion will cover the stages patients go through: **Testing for blood pressure (“screening and diagnosis”), starting treatment and taking treatment over time**. Please feel free to tell us whatever you are comfortable sharing. You should also remember that you do not have to share anything that you are not comfortable sharing. We will not write down your name. There are no right or wrong answers, so please be honest and tell us what is true for you and your community. If at any point during the discussion, you decide to leave, you are free to do so. This will also not incur any penalty. Are you ready to begin?”

**Step 4:** *Complete the fields below and then start with the FGD. Start by reiterating the importance of confidentiality within the group. As you ask the questions, please probe appropriately to gain as much depth on the topics as possible.*

**Facilitator name** ____________________ **Note-taker name ________________**

**Date (dd/mm/yyyy)** ____/ ____/ 20___

**Facility ___________________________**

**Time Start** _____: ____ **Time Finish** _____: _____

**Supervisor name ___________________________**

**0 Warm-up**

- 1. What role do you each play in hypertension care?

(go round to get brief descriptions from each FGD participant to help you understand the group)

1. **Screening and diagnosis**

*Let us first talk about new hypertension cases, how they are identified through screening and follow-up tests.*

How does the oblast find new cases in general or antenatal care?

***First*** *allow administrators to respond.* ***Then probe for:***

- How are new hypertension cases found?
- What is the role of the provider versus facility versus oblast-level administrator in finding new cases?
- What do you think works well in blood pressure screening/hypertension diagnosis?
- What do you think does not work well in identifying people with hypertension?
- Are all health staff trained on such guidelines? Does the oblast provide support for training and retraining of staff on screening for hypertension?
- Does the oblast provide any other support for hypertension screening, outside training?
- Is coverage of blood pressure screening monitored, reported, or rewarded? If yes, please describe how?
- What factors prevent people from having their blood pressure checked in your oblast?
- What resources exist in the facility and the community to support new hypertension cases?
- How can the oblast increase the proportion of the general and pregnant population who are screened for hypertension?

1. **Treatment start (initiation)**

What are your views on how hypertension treatment is started in new patients?

***First*** *allow administrators to respond.* ***Then probe for:***

- What role does the oblast versus facility have in ensuring that new cases of hypertension commence treatment?
- What works well in the current system of hypertension treatment initiation?
- What does not work well?
- Are all health staff trained on treatment guidelines? Does the oblast provide support for training and retraining of staff on treatment of hypertension?
- Does the oblast provide any other support for hypertension treatment, outside training?
- Is the initiation of treatment for hypertension following diagnosis monitored, reported, or rewarded? If yes, please describe how?
- What could prevent new cases from starting hypertension treatment?
- Do some people start treatment and stop? Why?
- How can the oblast increase the proportion of the general and pregnant population who start treatment for hypertension after screening?

1. **Treatment maintenance and monitoring**
   1. What do you think about the long-term care for hypertension patients in your oblast?

***First*** *allow administrators to respond.* ***Then probe for:***

- How does it work once somebody is on long-term treatment? [“maintenance phase”]
- What is the role of the oblast versus facility in supporting maintenance of hypertension treatment?
- What works well for patients in long-term hypertension care?
- What are important challenges for patients in long-term hypertension care?
- What are the reasons patients stop taking their hypertension drugs?
- Are there specific issues with ANC clients discontinuing hypertension treatment? [understand transition between ANC and adult hypertension care!]
- What support does the oblast give providers and patients to help with maintaining treatment?
- How can the oblast increase the proportion of the general and pregnant population who maintain treatment for hypertension?
  1. What are your views on patients “playing their part” in hypertension care?

***First*** *allow administrators to respond.* ***Then probe for:***

- Are patients empowered to self-monitor their hypertension treatment? With knowledge/risk perception? With blood pressure monitors? With supporting interventions like nutrition counselling, stress management, etc?
- What community support do patients receive? From the oblast? Support groups? NGOs? FBOs?
- Do ANC clients get any specific support?
  1. What do the health services do to promote treatment adherence among their hypertension patients and help them stay in care?

***First*** *allow administrators to respond.* ***Then probe for:***

- Facility supported strategies (e.g. Buddy system, text reminders, alternative places to pick up meds, longer prescriptions, triage into chronic track, counselling, timing of counselling, tracing)
- System strategies like integration (one-stop-shop drug), subsidies/co-payment, insurance, benefits
- Any additions on adherence support interventions in ANC?
- What else can the oblast do to promote treatment adherence?

1. **Primary prevention**
   1. How can Tajikistan prevent people from having high BP in the first place?

**Probe for:**

- Individual/family strategies?
- Health services strategies?
- Community strategies?
- Which strategies will work best for adults? For children? For ANCs?
- Is stigma (of obesity or of diabetes) an issue? What is needed to address stigma?
- If prevention strategies are not working, why not?
- What would makes them more effective?

*(Write these down on a flipchart)*

***Thank the participants for their time and contribution. Remind them that if any of them wish to share anything in private they can come to talk with you separately.***

***Close the meeting and complete the time the discussion finished on the first page.***

***Ask the supervisor to check consent papers, the demographic table, the notes and the auto-recording, and to sign on the first page. The supervisor is responsible for safekeeping of these items, once handed over.***
