# Supplementary material for: Challenges and opportunities in the continuity of care for hypertension: a mixed-methods study embedded in a primary health care intervention in Tajikistan
Source: BMC Health Serv Res. 2019 Dec 3;19:925. doi: 10.1186/s12913-019-4779-5 (PMC6889695; doi:10.1186/s12913-019-4779-5)
Supplement: Supplementary file 3 — Additional file 3. Focus Group Discussion Guide for Hypertension Care (Providers). A document describing eligibility for participation, instructions for the interviewer, and discussion prompts. [file 12913_2019_4779_MOESM3_ESM.docx]

**Hypertension Control Cascades**

Focus Group Discussion Guide for Hypertension Care (Providers)

Target Audience: FGDs to be conducted with **providers in** **health facilities** associated with the selected **study villages**

Eligibility: Providers of care in government health facilities including ANC services

**Instructions for the Interviewer**

**Step 1: Informed Consent**

*Ask each participant for about half an hour of their time. Introduce yourself and the study. Begin the informed consent as per the training. Leave the informed consent sheet with each participant. If consent is granted for FGD participation* ***and*** *audio-recording, proceed to next step*

**Step 2***:* **Identification of consented participant (continue from here on a set date, afternoon hours in a clinic, allowing for 2 hours of discussion)**

*Complete the basic demographic table for each consented participant as they sign in.* *This form will have an ID number for each participant. Make sure that the note taker has the correct ID numbers recorded on his/her notes prior to beginning and that participants have their correct “Letter Label” hanging around their neck to help the note taker with identification of participants.*

**Was written informed consent obtained for each participant before the start of the FGD (including permission by each to audio record this discussion)?**

**YES ________ (proceed with FGD)**

**NO** ________ (**STOP and only continue with the individuals who have consented to participate)**

**Step 3***:* **Introduction**

*Read the following statement:*

“Thank you for agreeing to participate in this discussion. My name is _______________. I will be asking you the questions. My partner _______________ will be taking notes on the things you have to say.

We want to understand your views on hypertension care provided by the public health sector. Our discussion will cover the stages patients go through: **Testing for blood pressure (“screening and diagnosis”), starting treatment and taking treatment over time**. Please feel free to tell us whatever you are comfortable sharing. You should also remember that you do not have to share anything that you are not comfortable sharing. We will not write down your name. There are no right or wrong answers, so please be honest and tell us what is true for you and your community. If at any point during the discussion, you decide to leave, you are free to do so. This will also not incur any penalty. Are you ready to begin?”

**Step 4:** *Complete the fields below and then start with the FGD. Start by reiterating the importance of confidentiality within the group. As you ask the questions, please probe appropriately to gain as much depth on the topics as possible.*

**Facilitator name** ____________________ **Note-taker name ________________**

**Date (dd/mm/yyyy)** ____/ ____/ 20___

**Facility ___________________________**

**Time Start** _____: ____ **Time Finish** _____: _____

**Supervisor name ___________________________**

**0 Warm-up**

- 1. What role do you each play in hypertension care?

(go round to get brief descriptions from each FGD participant to help you understand the group)

1. **Screening and diagnosis**

*Let us first talk about new hypertension cases, how they are identified through screening and follow-up tests.*

- 1. . How does this health facility go about finding new cases in general care (not ANC)?

***First*** *allow providers to respond.* ***Then probe for:***

- How are new hypertension cases found in general?
- What do you think works well in blood pressure screening/hypertension diagnosis?
- What do you think does not work well in identifying people with hypertension?
- Do you have hypertension care guidelines? Is all health staff trained on such guidelines? Are there opportunities to retrain on the use of these guidelines?
- Is everybody who does blood pressure testing trained on correct procedures?
- After one high blood pressure measurement, are suspected cases re-measured to see if there is hypertension? If so, when does this occur? When is the case referred and why or why not?
- What could put people off from having their blood pressure checked at the health service? Is there avoidance/refusal by patients to know they may have a blood pressure problem?
- Any views on combining of blood pressure screening with other services?
- Is health information provided during screening or after diagnosis?
- If new cases have questions about hypertension, what is available to them? (e.g. resources, materials, counselling staff?) In what format and language?
- Comments on the roles of the different health staff involved in diagnosis?
- What specific actions can health facilities take to increase screening for hypertension in the community? What constraints do health facilities face to adopting these actions?
  1. Given our interest in women in ANC, do you have comments about finding hypertension cases in ANC through blood pressure screening?

***First*** *allow providers to respond.* ***Then probe for:***

- In ANC, what do you think works well in hypertension screening/diagnosis? [not routine BP of known cases but finding new]
- In ANC, what do you think does not work well in identifying women with hypertension?
- What is particular to ANC services when it comes to finding new hypertension cases? [get at any differences compared to general adult care]
  1. How could hypertension diagnosis be improved, in adults in general care or in ANC?

***First*** *allow providers to respond.* ***Then probe for:***

- Is the health information at diagnosis patient- specific, do they meet the needs of *individuals*? If yes, describe how?
- How can the health services prevent pre-treatment loss to follow-up (people getting diagnosed with hypertension but not advancing to treatment)
- What concrete improvements could be made in the service offer for hypertension diagnosis? (seek specific detail on e.g. counselling content, referral) *(Write these down on a flipchart, separately for general adults and for ANC clients)*

1. **Treatment start (initiation)**
   1. What are your views on how hypertension treatment is started in new patients?

***First*** *allow providers to respond.* ***Then probe for:***

- What treatments are offered? Fixed-dose combinations vs. multiple individual drugs? Counselling/advice? Weight loss program? Diet education? Smoking cessation? Is there any patient choice?
- What works well in hypertension treatment initiation?
- What does not work well?
- Anything specific to getting hypertension treatment started in new cases in ANC?
- When are new cases offered hypertension treatment?
- Are treatment guidelines comprehensive on these options and clear? What information is missing, if any? What aspect of the guidelines could be clearer, if any?
- Are the right drugs always available? Are prescriptions given to a new case, instead of the drug itself?
- How much time do health staff spend with new hypertension patients? [length of consultation slots]
- What could prevent new cases from starting hypertension treatment?
- Do some people start treatment and stop? Why?
- What information is provided to the patient at treatment start? What topics are covered?
- If new cases have questions about hypertension treatment, what is available to them? (e.g. resources, materials, staff, contact numbers)
- Comments on polypharmacy (dug labelling, clarity of regimens, ease of prescriptions/refills)
- Comments on the roles of the different health staff involved in hypertension treatment initiation?
  1. How could the starting of hypertension treatment be improved, in adults in general care or in ANC?

***First*** *allow providers to respond.* ***Then probe for:***

- Does the counselling at hypertension treatment initiation meet the needs of *individuals*?
- What do new hypertension patients need most when they start their treatment?
- What concrete improvements could be made at the stage of starting hypertension treatment? (seek specific detail on e.g. IEC on drug’s adverse effects, drug refill, fixed dose combinations, etc.) *(Write these down on a flipchart, separately for general adults and for ANC clients)*

1. **Treatment maintenance and monitoring**
   1. What do you think about the long-term care for hypertension patients provided in your health facility?

***First*** *allow providers to respond.* ***Then probe for:***

- What services do you provide to somebody who is on long-term treatment? [“maintenance phase”]
- What works well for patients in long-term hypertension care?
- What are important challenges for patients in long-term hypertension care?
- Any specific observations on ANC clients in hypertension care?
- Are there specific issues with hypertension patients during their first weeks of treatment? [“consolidation phase”]
- What are the reasons patients stop taking their hypertension drugs?
- Are there specific issues with ANC clients discontinuing hypertension treatment? [understand transition between ANC and adult hypertension care!]
- Once on treatment, do healthcare workers support patients in specific ways to help with sticking to the treatment? What services are offered? Do they ever call? Give education? Ask you/remind about your medication?
- How helpful are the guidelines/protocols regarding long-term care?
- Adherence to non-pharma treatment (healthy eating, weight loss, physical activity, smoking cessation)?
- What are the views on clinic visit schedules and monitoring tests? [could probe on opening times, queues for chronic patients, getting an appointment, convenience of clinics for consultations, lab tests, lab results, drug refill]
- Do patients know when treatment is working well, or not working well? (communication of reaching/failing BP target, but also meeting targets cholesterol, weight/BMI, glucose)
- Perceptions of efficiency and convenience of long-term hypertension care? [is it a one-stop-shop]
- Comments on the roles of the different health staff involved in long-term care?
  1. What are your views on patients “playing their part” in hypertension care?

***First*** *allow providers to respond.* ***Then probe for:***

- Are patients empowered to self-monitor their hypertension treatment? With knowledge/risk perception? With blood pressure monitors? With supporting interventions like nutrition counselling, stress management, etc?
- Do they get support from other structures like support groups, NGOs/FBOs?
- Any additions on ANC clients?
  1. What do the health services do to promote treatment adherence among their hypertension patients and help them stay in care?

***First*** *allow providers to respond.* ***Then probe for:***

- Facility supported strategies (e.g. Buddy system, text reminders, alternative places to pick up meds, longer prescriptions, triage into chronic track, counselling, timing of counselling, tracing)
- System strategies like integration (one-stop-shop drug), subsidies/co-payment, insurance, benefits
- Any additions on adherence support interventions in ANC?
  1. What do health facilities do to specifically support patients who fail their BP target while on treatment?

**Probe for:**

- Increased BP monitoring intervals?
- Diagnosis of root-cause of non-adherence? Specific counselling/IEC?
- Treatment step-up?
- Specific staff training/focal point?
- What else would work for these patients who struggle to reach BP target? Anything specific for ANCs?
  1. How could long-term hypertension care be improved?

**Probe for:**

- Is the care provided sufficiently patient-centered/individualized?
- Are test results used and communicated as part of patient care?
- What could health facilities do differently to promote adherence?
- Are there changes needed to make these work well in the community? What? Why?
- Do different groups need different support? ANCs?
- What else is needed? What other support would help?

*(Write these down on a flipchart)*

1. **Primary prevention**
   1. How can Tajikistan prevent people from having high BP in the first place?

**Probe for:**

- Individual/family strategies?
- Health services strategies?
- Community strategies?
- Which strategies will work best for adults? For children? For ANCs?
- Is stigma (of obesity or of diabetes) an issue? What is needed to address stigma?
- If prevention strategies are not working, why not?
- What would makes them more effective?

*(Write these down on a flipchart)*

***Thank the participants for their time and contribution. Remind them that if any of them wish to share anything in private they can come to talk with you separately.***

***Close the meeting and complete the time the discussion finished on the first page.***

***Ask the supervisor to check consent papers, the demographic table, the notes and the auto-recording, and to sign on the first page. The supervisor is responsible for safekeeping of these items, once handed over.***
